# P-473. Health Care Resource Use Burden Among People With HIV (PWH) and Concurrent Mental Health Disorders (MHDs): Claims Analysis of Treatment-Naïve Medicaid Population Initiating Multi-Tablet Regimens (MTRs) vs. Single-Table Regimens (STRs)

**DOI:** 10.1093/ofid/ofae631.672

**Published:** 2025-01-29

**Authors:** Megan Chen, Fatema A Turkistani, Mary J Christoph, Seojin Park, Woodie Zachry, Amy Weinberg, Cassidy Trom, Joshua Gruber, Krithika Rajagopalan

**Affiliations:** Gilead Sciences, Inc., Foster City, California; Anlitiks, Inc., Dover, Massachusetts; Gilead Sciences, Inc., Foster City, California; Gilead Sciences, Inc., Foster City, California; Gilead Sciences Inc, Foster City, California; Gilead Sciences, Inc., Foster City, California; Gilead Sciences, Inc., Foster City, California; Gilead Sciences, Forest City, California; Anlitiks, Inc., Dover, Massachusetts

## Abstract

**Background:**

Multi-tablet antiretroviral (ART) regimens (MTRs) have been largely replaced by single-tablet ART regimens (STRs) for treating people with HIV(PWH). STRs provide lower pill burden, increased patient adherence, and lower health care resource utilization (HCRU) among PWH. However, their effects among vulnerable sub-populations of PWH with coexisting mental health disorders (PWH/MHDs) in real-world settings are not well understood. This analysis examined the characteristics and HCRU patterns among PWH/MHDs who initiated MTRs vs STRs in a US Medicaid population.

**Methods:**

A retrospective analysis of treatment-naïve Medicaid PWH/MHDs (based on claims for MHD-related diagnosis or treatments) using Anlitiks All Payor Claims database from 01/01/16-06/30/23 was conducted. The study included MTR or STR initiators (i.e., MTR/STR initiation date=index date) with ≥12-months pre- and post-index continuous enrollment. Baseline characteristics and post-index all-cause HCRU outcomes (PWH/MHD with ≥1 inpatient (IP) hospitalization, outpatient (OP) visits, emergency department (ED) visits, office visits, and other visits) were described using Chi-square, t-tests and Wilcoxon rank-sum tests as appropriate. HCRU outcomes were assessed using multivariate logistic regression models and reported as adjusted odds ratios (aOR) and 95% confidence intervals (CI).

**Results:**

Among PWH/MHDs, MTR initiators (n=7,874) vs. STR initiators (n=46,024) were younger (43.6 vs. 47.2 years; p< 0.05), more likely to be female (64.3% vs. 53.6%; p< 0.05), and more likely to use substances (32.1% vs 29.4%; p< 0.05). MTR initiators had significantly higher aOR for: IP hospitalizations (1.49; CI:1.41-1.56), ER visits (1.38; CI: 1.31-1.44), OP visits (1.19; 1.44-1.26), and other visits (1.19; 1.44-1.26) compared to STR initiators. However, MTR initiators had significantly lower aOR (0.94; 0.89-0.99) for office visits compared to STR initiators.

**Conclusion:**

This analysis indicates that PWH with MHDs in the Medicaid population are more likely to have higher HCRU while taking MTR compared to STR. The analysis also highlights that PWH with MHDs may be the underserved population who may benefit from receiving more tailored and simplified ART regimens.

**Disclosures:**

**Megan Chen, MSPH**, Gilead Sciences, Inc.: Honoraria|Gilead Sciences, Inc.: Employee|Gilead Sciences, Inc.: Stocks/Bonds (Private Company) **Mary J. Christoph, PhD, MPH**, AstraZeneca: Advisor/Consultant|AstraZeneca: Former employee|AstraZeneca: Stocks/Bonds (Private Company)|Gilead Sciences, Inc.: Employee|Gilead Sciences, Inc.: Stocks/Bonds (Private Company) **Seojin Park, PharmD, MS**, Gilead Sciences, Inc.: Employee|Gilead Sciences, Inc.: Stocks/Bonds (Public Company) **Woodie Zachry, RPh, PhD**, Gilead Sciences, Inc.: Employee|Gilead Sciences, Inc.: Stocks/Bonds (Public Company) **Amy Weinberg, DNP, MS**, Gilead Sciences, Inc.: Employee|Gilead Sciences, Inc.: Stocks/Bonds (Private Company) **Cassidy Trom, PharmD, AAHIVE**, Gilead Sciences, Inc.: Employee|Gilead Sciences, Inc.: Stocks/Bonds (Private Company) **Joshua Gruber, PhD MPH**, Gilead Sciences, Inc.: Employee|Gilead Sciences, Inc.: Stocks/Bonds (Public Company) **Krithika Rajagopalan, PhD**, Gilead Sciences, Inc.: Advisor/Consultant

